# Time-Based and Path-Based Analysis of Upper-Limb Movements during Activities of Daily Living

**DOI:** 10.3390/s23031289

**Published:** 2023-01-23

**Authors:** Sebastjan Šlajpah, Eva Čebašek, Marko Munih, Matjaž Mihelj

**Affiliations:** Faculty of Electrical Engineering, University of Ljubljana, Tržaška cesta 25, 1000 Ljubljana, Slovenia

**Keywords:** stroke, upper-limb movement, movement estimation, activities of daily living, inertial measurement unit, electromyography

## Abstract

Patients after stroke need to re-learn functional movements required for independent living throughout the rehabilitation process. In the study, we used a wearable sensory system for monitoring the movement of the upper limbs while performing activities of daily living. We implemented time-based and path-based segmentation of movement trajectories and muscle activity to quantify the activities of the unaffected and the affected upper limbs. While time-based segmentation splits the trajectory in quants of equal duration, path-based segmentation isolates completed movements. We analyzed the hand movement path and forearm muscle activity and introduced a bimanual movement parameter, which enables differentiation between unimanual and bimanual activities. The approach was validated in a study that included a healthy subject and seven patients after stroke with different levels of disabilities. Path-based segmentation provides a more detailed and comprehensive evaluation of upper limb activities, while time-based segmentation is more suitable for real-time assessment and providing feedback to patients. Bimanual movement parameter effectively differentiates between different levels of upper limb involvement and is a clear indicator of the activity of the affected limb relative to the unaffected limb.

## 1. Introduction

Rehabilitation is a process of restoring physical, sensory, and mental capabilities lost due to injury, disease, illness, or other conditions, congenital or acquired. With the development of medicine and, consequently, humanity ageing, the need for rehabilitation due to various diseases rises permanently [1,2].

When patients after stroke are discharged from rehabilitation facilities, they usually still have some degree of upper limb impairment. Therefore, the therapy should be an ongoing process that continues after the patient leaves the rehabilitation institution. Active use of an affected limb to perform various movements has a positive effect on motor abilities. Daily activities with the affected limb are essential for faster improvement [3]. Therefore, patients should use their affected upper limbs as much as possible during daily tasks. Monitoring the patient in the home environment while performing daily activities is one of the critical goals in rehabilitation [4,5]. These measurements can help identify motor activities [6], determine the frequency of individual activities and the relation between bimanual and unimanual actions, compute the level of use of a particular body segment [7], assess the limb usage duration and movement quality [8], and identify limitations in performing the activities.

In robotics, neuroscience, and ergonomics research, parameters computed from the limb’s movement trajectory are often used [9]. We can obtain information about the movement’s quality, speed, smoothness, and the distance travelled from the movement trajectory [10]. We can use different parameters for trajectory comparison, for example, Euclidean distance, Dynamic Time Warping, Longest Common Subsequence based measures, Hausdorff, or Fréchet distance [11,12,13,14]. Researchers also focus on movement repeatability and the limb final position [15].

Rehabilitation training exercises and assessment of motor abilities are typically based on various sensing technologies [16]. Researchers use optoelectronic systems to measure movement in the laboratory environment, ensuring high accuracy of the obtained results. Due to the high cost and limited measuring range of these systems, inertial measuring units (IMUs) have gained popularity. They represent a low-cost alternative [17]. Since sensors are small and portable, they do not restrict the subject’s movement or range of motion [18]. Wearable sensors are also not limited to a laboratory environment or rehabilitation facility, meaning the subject can wear them at home during daily activities.

In addition to position sensors, muscle activity or electromyographic (EMG) sensors are also used to assess movement [19,20,21]. In robotics, EMG signals are often used to guide robots [22] or control the active prosthesis [20]. For research outside the laboratories, we can also use wearable sensors, such as the MYO bracelet (Thalmic Labs) [23], which detects electrical activity on the skin’s surface when the muscles of the forearm contract. From these signals, we can extract information about the movement of fingers, which allows for detecting the grip [24]. We can also indirectly estimate the grip force, which can replace a force sensor in some tasks [25].

With advancements in technology, particularly MEMS, the miniaturization of wearable sensors has taken a significant leap forward. IMUs, for example, are now compact and lightweight, measuring just a few centimeters in size and weighing around 10 grams with the battery included [26,27]. When packaged in a suitable housing, they make for an ideal sensor system for measuring motion, as they are small, easy to use, and will not impede the user’s movement. The same can be said for electromyography measurements, which have transformed from bulky laboratory equipment with wires and electrodes to simple wristbands with built-in electrodes, making it more widely accessible. Furthermore, smart wristbands now offer a combination of sensor modalities such as inertial measurements, the electromyogram, the photoplethysmogram, electrodermal activity signal, and the skin temperature signal [28]. In the era of IoT, sensors have the capability to both store data locally on the device and send them to the cloud for further processing. Additionally, connectivity with mobile phones is crucial for logging and real-time processing of data, as well as serving as an additional sensor module.

Inertial measurement units and electromyography measuring devices are low-cost and widely used due to their easy availability. The development and utilization of new nanomaterials in novel structures have the potential to expand the range of wearable sensors [29,30]. While the use of wearable sensors is promising, it is currently limited by their difficult and expensive production.

In this paper, we propose and validate a methodology for analysing continuous movement trajectories acquired during activities of daily living. We compare time-based and path-based segmentation of movement trajectories into relevant quants and introduce several parameters for assessing upper limb functions. The upper limb bimanual movement parameter is introduced to distinguish between unimanual and bimanual movements during the activities of daily living.

## 2. Methodology

During rehabilitation, patients perform various tasks that imitate the basic movements in many daily activities. After the rehabilitation, the patient should continue using the affected limb during activities of daily living to further improve the affected limb’s capabilities. However, in the home environment, patients often lack the motivation to use their affected limb to the same extent as in therapy and learn to perform basic activities only with the unaffected limb, thereby inhibiting the improvement of the affected limb’s movement skills. Therefore, measurement of daily activities is vital for the continuous assessment of the affected upper limb use and subsequent rehabilitation program.

**Figure 1:**
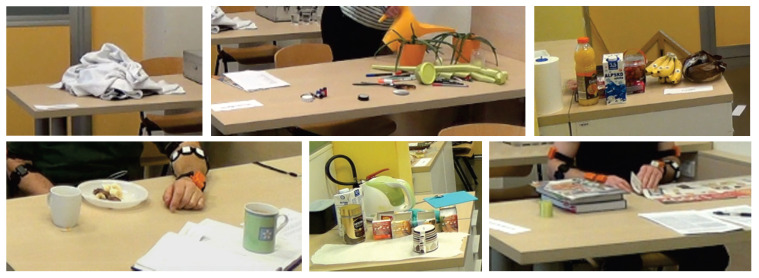
Activities of daily living included preparing a meal, chatting and drinking coffee or tea, watering flowers, folding and tidying towels, writing a shopping list, reading a newspaper, and tidying up a desk.

In this study, we measured activities of daily living in a home-like environment simulated in a laboratory setting. We prepared the tasks that consisted of activities of daily living (Figure 1): preparing a meal, chatting and drinking coffee or tea, watering flowers, folding and tidying towels, writing a shopping list, reading a newspaper, and tidying up a desk. The listed activities were presented to each participant. We stressed that they should perform the tasks as they would at home and not have to make an effort to use the affected upper limb as they would in therapy.

### 2.1. Participants

The study included seven post-stroke patients (four males) aged 44–59 years (average 51.6 years) and 5–80 weeks after stroke (average 25 weeks), with limited upper limb usage. Three participants had left upper limb hemiparesis, and the rest had right upper limb hemiparesis. All patients were right-handed before the stroke. One healthy, 26 years old male was included in the study, providing reference values for interpreting patients’ results. We acquired approximately 15 hours of daily activities.

All the participants signed the informed consent. The study was approved by the National medical ethics committee of the Republic of Slovenia (80/03/15).

### 2.2. System for Measurement of Daily Activities

Upper limb movement was measured with a wearable sensory system consisting of seven wireless IMU sensors and two EMG armbands (MYO armbands from Thalmic labs), more thoroughly described in a study to quantify movement during Action Research Arm Test (ARAT) on a group of patients after stroke [31]. The IMU sensor consists of a tri-axial gyroscope with a measuring range of $\pm 1000^{\circ }$/s and a sampling rate of 1 kHz, a tri-axial accelerometer with a measuring range of ±2 g and a sampling frequency of 1 kHz, and a tri-axial magnetometer with a measuring range of ±130 μT and a sampling frequency of 160 Hz. The sampling and transmission of the seven IMUs occurred at a frequency of 80 Hz. The accuracy of the measured IMU angle is below $2.5^{\circ }$ for stationary conditions and bellow $5^{\circ }$ under dynamic conditions. The Myo bracelet (Thalmic Labs), consists of eight electromyographic electrodes evenly distributed around the inner circumference of the bracelet. The data are sampled at a rate of 200 Hz.

The system is designed for the measurement of long-duration activities of daily living. Data are acquired on a stick computer. We provided warning signals in case of malfunctions, such as the USB receiver not being inserted, the IMU being off or not working, or the IMU’s battery being empty. We used built-in vibration and a signalling light on the EMG bracelets to make warning signals unambiguous.

**Figure 2:**
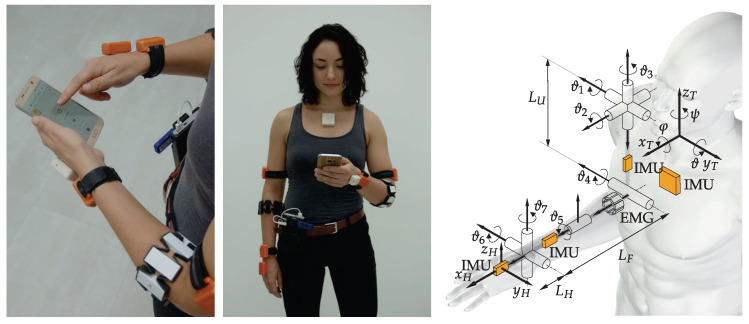
Wearable system for measurement of activities of daily living and kinematic model of the upper limb with sensor placement: IMU sensors (orange) and EMG bracelets (white segmented cylinder).

Communication between the stick computer and the researcher in charge is enabled through an HTML application for a mobile phone. When the measurement is finished, the application stores the results in a separate folder on the computer. The wearable measuring system shown in Figure 2 has several advantages: (1) it is not limited to the laboratory setting, and it can be used in a home environment or outdoors, (2) the use of the system only requires a mobile phone with enabled WiFi connectivity, (3) the sensors are physically small and unobtrusive during upper limb movement, and 4) the system allows movement measurement for up to six hours. In Figure 2 (right), orange boxes represent IMU sensors, and the segmented cylinder represents armband EMG electrodes. The trunk, which represents the reference frame, and the two arms are relevant to the analysis. The posture in Figure 2 (right) defines zero values for all joint angles. In the presented kinematic model, the arm joints are defined in the following order from proximal to distal: (1) shoulder flexion/extension, (2) shoulder abduction/adduction, (3) shoulder internal/external rotation, (4) elbow flexion/extension, (5) wrist pronation/supination, (6) wrist ulnar/radial deviation, and (7) wrist flexion/extension.

### 2.3. Movement Segmentation

The hand position is defined relative to the trunk reference coordinate frame $\left(x_{T},y_{T},z_{T}\right)$ shown in Figure 2. The hand trajectory p(t) is the path p that the hand follows through space as a function of time *t*. While measuring activities of daily living, the trajectory can be segmented into submovements using different approaches. A trivial approach is to segment the trajectory into predefined time intervals where movement parameters are then computed on these shorter intervals. We will refer to this approach as a time-based segmentation. A more complex approach is to segment long trajectories into completed submovements [32]. We will refer to this approach as a path-based segmentation. In general, speed and form can be separated by parametrization with the arc length [33]. With path-based movement segmentation, the entire trajectory of the arm p(t) can be divided into shorter movement intervals by first representing it as a function of arc length. With the path-based segmentation method, more thoroughly described in [31], we split a continuous trajectory into *M* discrete movements.

To validate the path-based segmentation method, we completed a test with a healthy subject sitting at a table and a sheet of paper with regular hexagons printed on it. The person traced the sides of a printed hexagon with a pencil. This task was repeated with hexagons with different base side lengths (20 mm, 50 mm, and 100 mm). The hand speed varied during execution. The upper limb kinematics was calculated relative to the trunk. We counted the number of times the subject drew a straight line across the printed side of the hexagon for later comparison with the automatic path-based segmentation of motion during the drawing. The angle between the adjacent sides of a regular hexagon is $120^{\circ }$, which was used to verify the detection of a change in motion direction (one of the criteria for movement segmentation). The 20 mm side length of the hexagon is at the resolution limit of the used measurement system ($2.5^{\circ }$ measurement error in the shoulder joint represents an error in the order of 20 mm at the finger position when the arm is extended). For the purpose of segmenting activities of daily living in this study, the current resolution of the measurement system is sufficient. However, the analysis of more subtle movements, such as writing, may require a higher precision measurement system such as an optoelectronic system.

**Figure 3:**
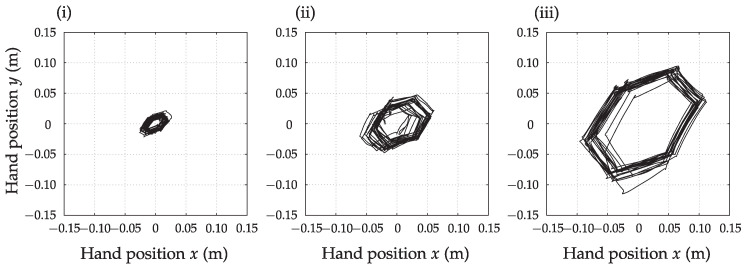
The traversed path of the hand during the sequential drawing of the circumference of a regular hexagon with the length of the base side (**i**) 20 mm, (**ii**) 50 mm, and (**iii**) 100 mm.

Figure 3 shows the previously described movement in the transverse plane. The path-based movement segmentation method enables the setting of minimal movement length to be detected. Movement along the circumference of the hexagon with the smallest side length (20 mm) is shown in Figure 3 (i), where the subject made 186 movements. The movement segmentation method yielded a result of M=172. The system detected fewer movements because the path length was at the limit of the system resolution. The measurement with a hexagon base length of 50 mm is presented in Figure 3 (ii), where the subject performed 120 movements, and the path-based segmentation method obtained a result of M=121. The same was repeated for the longest hexagon base length 100 mm with trajectory represented in Figure 3 (iii). Here the subject performed 120 movements, and the path-based segmentation method returned a result of M=124. The system detected some more movements than were performed as the drawn lines were not perfectly straight and continuous. Nevertheless, the segmentation error is at the level of a few percent, which is enough for automatic path-based segmentation of continuous movements during activities of daily living. To analyze the movement of the upper limbs during daily activities, we defined a minimum movement length of 50 mm to emphasize activities with longer arm trajectories.

### 2.4. Analysis of Upper Limb Activities with Time-Based Segmentation

Studies that use accelerometers to analyze upper limb movement often rely on the established activity counts parameter for the analysis [34,35,36,37,38,39,40]. The parameter is defined as the sum of the accelerations within a specified time interval, which is not prescribed but is usually selected in the time frame of up to two seconds. The parameter can distinguish between the more active and the less active upper limb. The parameter is determined by summing the accelerations over an unspecified but typically short time period, such as within two seconds. This parameter allows for differentiation between higher and lower levels of upper limb activity.

Each subject performed approximately two hours of activities of daily living that were measured. We set T=0.25 s ($T_{i}\;=\;T_{i-1}+T$) for the segment duration. In the following equations, the notation ${}_{\left(AF,UAF\right)}$ indicates that the same equation applies to both limbs, with ${}_{UAF}$ for the unaffected and ${}_{AF}$ for the affected limb. First, we computed the activity counts AC for the unaffected (UAF) and affected (AF) upper limbs in the time interval *i* as

(1) $$AC_{{\left(AF,UAF\right)}_{i}}^{h}=\frac{1}{T}\int_{T_{i-1}}^{T_{i}}∥a_{h_{\left(AF,UAF\right)}}\left(t\right)∥dt,$$

where $a_{h}$ represents acceleration measured at the hand position and filtered using a bandpass filter with cut-off frequencies 0.25 Hz and 2.5 Hz [39]. The activity counts AC, a measurement of limb activity, is computed for each upper limb. Bandpass filtering does not entirely remove the gravitational acceleration. Thus we augmented the activity counts computation by subtracting gravitational acceleration g

(2) $$AC_{{\left(AF,UAF\right)}_{i}}^{\text{-}g}=\frac{1}{T}\int_{T_{i-1}}^{T_{i}}∥a_{h_{\left(AF,UAF\right)}}\left(t\right)-g∥dt.$$

The apparent movement of the arms may also be due to the movement of the trunk when the arm is at rest relative to the trunk. To exclude the influence of trunk movement, we introduced a third relation based on the subtracted trunk acceleration $a_{t}\left(t\right)$ (the subtraction also removes the effect of gravity)

(3) $$AC_{{\left(AF,UAF\right)}_{i}}^{\text{-}t}=\frac{1}{T}\int_{T_{i-1}}^{T_{i}}∥a_{h_{\left(AF,UAF\right)}}\left(t\right)-a_{t}\left(t\right)∥dt.$$

All acceleration vectors are expressed in the trunk coordinate system in the above equations.

Bailey et al. presented activity counts of both upper limbs $AC_{i}$ and the ratio of left, and right upper limb activity counts $R_{AC_{i}}$ [38,39]. Similarly, we summed values of activity counts of affected (non-dominant for the healthy subject) $AC_{AF_{i}}$ and unaffected (dominant for the healthy subject) $AC_{UAF_{i}}$ upper limb for each time interval

(4) $$AC_{i}=AC_{UAF_{i}}+AC_{AF_{i}}.$$

The parameter $AC_{i}$ represents the intensity of activities of both upper limbs, where 0 means that neither the affected (non-dominant) nor the unaffected (dominant) upper limb is active in the time interval. We evaluated activity count ratio $R_{AC_{i}}$ as

(5) $$R_{AC_{i}}=\ln\frac{AC_{UAF_{i}}+1}{AC_{AF_{i}}+1},$$

with which we prevent positive skewness coefficient of untransformed size ratios $R_{AC_{i}}>0$ [41].

**Figure 4:**
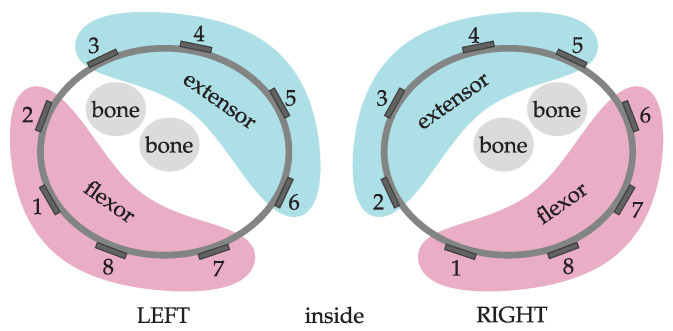
Muscles inside the forearm with the consequent electrodes numbers of EMG armband.

To analyze muscle activity resulting from time-based segmentation, we introduced muscle activity counts MC. We used signals from all eight electrodes of the EMG armband to estimate the total muscle activity. Four electrodes were assigned to each group to estimate the muscle activity of flexor and extensor muscle groups, as shown in Figure 4.

The muscle activity counts were computed from the adapted Equation (1) as

(6) $$MC_{{\left(AF,UAF\right)}_{i}}=\frac{1}{T}\int_{T_{i-1}}^{T_{i}}\sqrt{\sum_{N}w_{n}^{2}\left(t\right)}dt,$$

with T=0.25 s, $w_{n}$ is the muscle activity measured with electrode *n*, and *N* is the set of electrodes as determined in Figure 4 (all eight electrodes were considered for total muscle activity, N=8). We computed muscle activity counts of both upper limbs. Then we summed values of activity counts of unaffected (dominant) $MC_{UAF_{i}}$ and affected (non dominant) $MC_{AF_{i}}$ upper limb for each time interval to obtain

(7) $$MC_{i}=MC_{UAF_{i}}+MC_{AF_{i}}.$$

Finally, we evaluated the activity count ratio as

(8) $$R_{MC_{i}}=\ln\frac{MC_{UAF_{i}}+1}{MC_{AF_{i}}+1}.$$

### 2.5. Analysis of Upper Limb Activities with Path-Based Segmentation

In this case, the goal of movement analysis is not to identify specific everyday activities but to assess the movement capabilities of the upper limbs. Authors typically estimate kinematic parameters on submovements generated by time-based segmentation and analyze the movement as a function of time [42,43,44,45].

Activities of daily living consist of bimanual and unimanual movements. By comparing the number and the lengths of the movements of one and the other hand, we can determine which arm is more active in terms of movement frequency and amplitude. We based the assessment of movements of the upper limbs on four criteria combined into a single bimanual movement parameter BMP. The four criteria are: (1) the ratio of the lengths of the hands travelled paths $R_{L}$, (2) Pearson linear correlation coefficient PCC, (3) Fréchet distance *F*, and (4) variance ratio $R_{V}$.

We computed the hand path length $L_{{\left(AF,UAF\right)}_{m}}$ for the complete movement *m* resulting from path-based segmentation as

(9) $$L_{{\left(AF,UAF\right)}_{m}}=\int_{T_{o_{m}}}^{T_{t_{m}}}∥{\dot{p}}_{\left(AF,UAF\right)}\left(t\right)∥dt,$$

where *m* represents the index of the single completed movement within the continuous trajectory of arm movement and $\dot{p}$ represents velocity of the hand. The path length was calculated between the movement onset time $T_{o_{m}}$ and the movement termination time $T_{t_{m}}$ of the completed movement *m*. To compare the movements of the upper limbs, we calculated the natural logarithm of the ratio of the hand path lengths during the completed movement *m* as

(10) $$R_{L_{m}}=\ln\frac{L_{UAF_{m}}}{L_{AF_{m}}}.$$

The $R_{L_{m}}$ value should be close to zero for bimanual movements.

The Pearson linear correlation coefficient $PCC_{m}$ ([46]) and the Fréchet’s distance $F_{m}$ ([47]) were calculated between the speed profiles of the unaffected (dominant) and affected (non-dominant) limbs for the movement *m*. The value of $PCC_{m}$ is close to one in the case of bimanual movements. The Fréchet’s distance $F_{m}$ is a measure of similarity between two curves, which takes into account the position and sequence of points on the curve and is computed based on the definition in [12]. The Fréchet’s distance $F_{m}$ should be close to zero for bimanual movements.

Variance $V_{m}$ was computed as

(11) $$V_{{\left(AF,UAF\right)}_{m}}=\frac{1}{N_{t_{m}}-N_{o_{m}}-1}\sum_{k=N_{o_{m}}}^{N_{t_{m}}}{\left(∥{\dot{p}}_{{\left(AF,UAF\right)}_{k}}∥-\mu_{{\left(AF,UAF\right)}_{m}}\right)}^{2},$$

where $\mu_{m}$ is defined as

(12) $$\mu_{{\left(AF,UAF\right)}_{m}}=\frac{1}{N_{t_{m}}-N_{o_{m}}}\sum_{k=N_{o_{m}}}^{N_{t_{m}}}∥{\dot{p}}_{{\left(AF,UAF\right)}_{k}}∥,$$

${\dot{p}}_{k}$ represents the hand movement velocity at sample time *k*, $N_{o_{m}}$ represents the movement onset sample, and $N_{t_{m}}$ represents the movement termination sample. The ratio of variances used as one of the criteria for determining bimanual movement was computed as

(13) $$R_{V_{m}}=\ln\frac{V_{{UAF}_{m}}}{V_{{AF}_{m}}}.$$

The $R_{V_{m}}$ value should be close to zero for bimanual movements.

The bimanual movement parameter BMP is computed as

(14) $$BMP_{m}=1-\frac{1}{4}\left(\frac{1-PCC_{m}}{\alpha_{PCC}}+\frac{|R_{V_{m}}|}{\alpha_{R_{V}}}+\frac{F_{m}}{\alpha_{F}}+\frac{|R_{L_{m}}|}{\alpha_{R_{L}}}\right),$$

where denominator α indicates the weight of each criterion. The weights were selected to account for the 95 percentile of the measured parameters for all subjects and are $\alpha_{PCC}=2$, $\alpha_{R_{V}}=$ 5.5, $\alpha_{F}=1.3\;$ m, and $\alpha_{R_{L}}=2.5$. Parameter $BMP_{m}$ is normalized between 0 and 1 (1 for bimanual movements).

**Figure 5:**
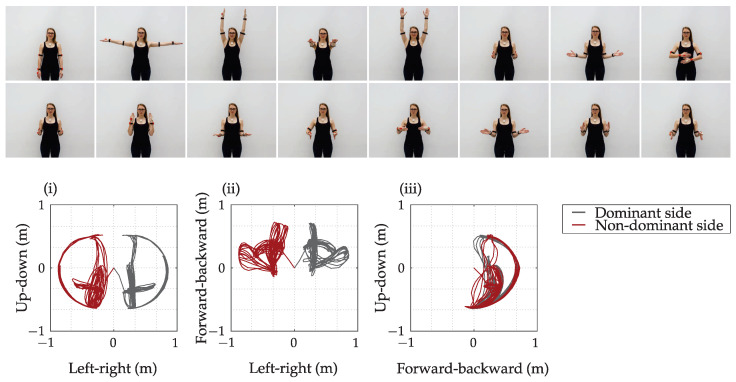
Symmetrical upper limb movement demonstration of a healthy subject with the corresponding trajectories of the arm in the frontal (**i**), transversal (**ii**) and sagittal (**iii**) plane. Black line represents the position of the dominant hand and red line represents the position of the non-dominant hand.

An example of a sequence of symmetric upper limb movements with the associated hand trajectories represented in three planes (frontal, transverse, and sagittal) is shown in Figure 5 for a healthy female subject. Arm trajectories were computed relative to the trunk coordinate system.

**Figure 6:**
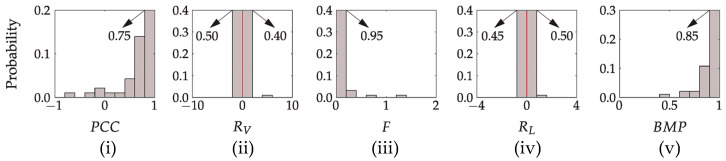
Probability distribution of movement parameters: (**i**) the linear correlation coefficient PCC, (**ii**) the ratio of variances $R_{V}$, (**iii**) the Fréchet distance *F*, (**iv**) the ratio of hand path lengths $R_{L}$, and (**v**) the upper limb bimanual movement parameter BMP of a healthy subject for the activities shown in Figure 5. The red vertical line marks the boundary between the dominant and non-dominant upper limb. The arrows indicate the peak values in each plot.

Figure 6 summarizes the movement parameters PCC, $R_{V}$, *F*, $R_{L}$, and BMP using the path-based segmentation corresponding to the symmetric movements of a healthy subject shown in Figure 5. All criteria, including bimanual movement parameter BMP, indicate well-coordinated movement between the upper limbs.

**Figure 7:**
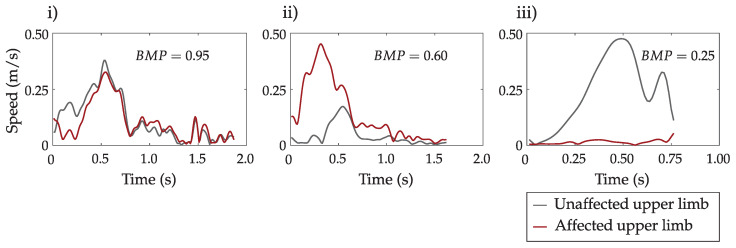
Representative examples of bimanual movements of a patient with an upper limb impairment for BMP=0.95 (**i**), BMP=0.60 (**ii**), and BMP=0.25 (**iii**). Black colour represents the speed of the unaffected arm and the red colour represents the speed of the affected arm.

To better represent different levels of movement coordination, Figure 7 shows the speed profiles of three representative movement cases of a patient with an upper limb impairment and the associated values of bimanual movement parameter BMP. We used the BMP values to distribute the upper limb movements into three classes. The BMP thresholds were set based on the visual analysis of hand movement trajectories. Mostly bimanual movements were defined with BMP>0.7, where coordination between arm activities was notable. Mostly unimanual movements were defined with BMP<0.4, where only one arm was active. Condition 0.4≤BMP≤0.7 indicates activities which cannot be strictly classified as bimanual or unimanual.

The average forearm muscle activity $W_{m}$ for each completed movement *m* was estimated separately for each upper limb based on the measured EMG signals, where we considered all eight electrodes of the EMG armband and computed the total value as

(15) $$W_{{\left(AF,UAF\right)}_{m}}=\frac{1}{T_{m}}\int_{T_{o_{m}}}^{T_{t_{m}}}\sqrt{\sum_{n=1}^{8}w_{n}^{2}\left(t\right)}dt,$$

where $w_{n}\left(t\right)$ represents value from electrode *n*. Then we determined the ratio of the muscle activity of the upper limbs as

(16) $$R_{W_{m}}=\ln\frac{W_{UAF_{m}}}{W_{AF_{m}}}.$$

## 3. Results

All participants performed the same tasks (Figure 1) and received the exact instructions about the measurement protocol. There was no specific order of activities of daily living, and the patient was allowed to skip the activity due to a reduced upper limb function. All patients’ movements were used in the later analysis. However, to reduce the number of figures, detailed results are presented only for one healthy subject and two post-stroke patients that differ in their upper limb movement capabilities (one with preserved upper limb functions and one with severe disability). Where applicable, a summary of movement parameters for all subjects is presented in a tabular form.

### 3.1. Time-Based Segmentation Movement Analysis

**Figure 8:**
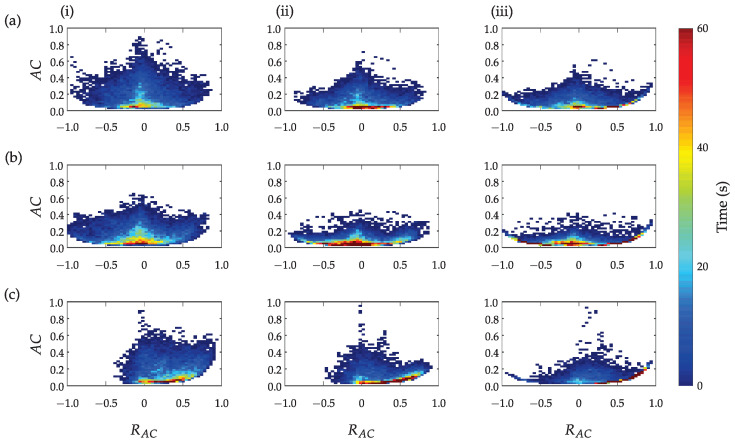
Upper limb activity counts AC in relation to $R_{AC}$ computed from accelerations (**i**), from accelerations with subtracted gravitational acceleration g (**ii**), and from accelerations with subtracted trunk acceleration (**iii**) for a healthy person (**a**) and two patients after stroke (**b**,**c**).

Figure 8 presents the activity counts computed based on three different approaches for the three subjects: (i) method (1) proposed by the authors in [38], (ii) method (2) with the subtracted gravitational acceleration (only dynamic acceleration as a result of subject’s activity), and (iii) method (3) with the subtracted trunk activity (only dynamic arm acceleration as a result of arm movement). All results are presented as a function of the ratio between the activity counts of the unaffected (dominant) upper limb with the affected (non-dominant) upper limb. Higher AC values indicate the higher intensity of bilateral activities, while the colour represents the total time in seconds for a given combination of parameters AC and $R_{AC}$. The three methods indicate similar activity counts symmetry for subjects (a) and (b) and asymmetry for the patient (c). However, the subtraction of gravitational or trunk acceleration reduces the overall movement intensity (indicated as lower AC peak values in plots (ii) and (iii)) and enables better expression of differences between arm movements.

**Figure 9:**
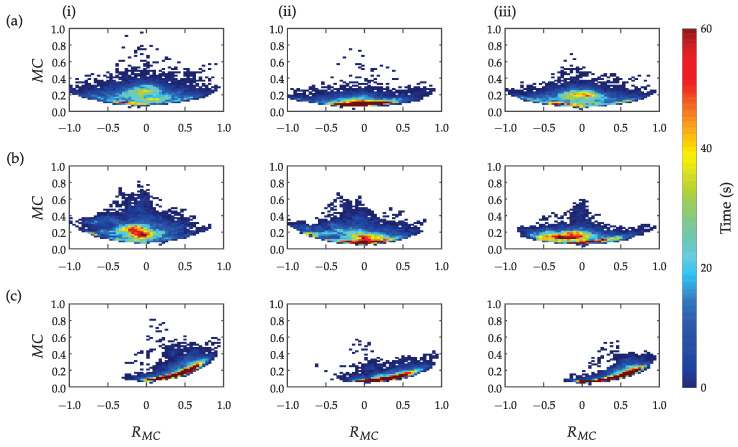
Upper limb muscle activity counts MC in relation to ratio $R_{MC}$ for total forearm flexor and extensor muscles (**i**), for forearm flexors (**ii**), and forearm extensors (**iii**) for a healthy person (**a**) and two stroke patients (**b**,**c**).

Figure 9 presents muscle activity counts MC in relation to the ratio $R_{MC}$. Figure 9 (i) represents the total forearm muscle activity, Figure 9 (ii) only for flexor muscles, and Figure 9 (iii) only for extensor muscles. Symmetric distribution can be noticed for the healthy subject and the patient with better preserved upper limb function. Significantly asymmetric activation was found for the patient with severe upper limb disability. Similar activation patterns were observed for the total activation and the activation of flexor and extensor muscles as shown in plots (i), (ii), and (iii). However, subtle differences can also be discerned between activation patterns. For example, longer low-level and symmetric activation of forearm flexor muscles compared to extensor muscles can be noted for the healthy subject. Notable similarities between the upper limb activity counts presented in Figure 8 and the muscle activity counts presented in Figure 9 can also be seen.

### 3.2. Path-Based Segmentation Movement Analysis

In the analysis of activities of daily living, we obtained a different number of movements *M* for each participant as a result of path-based movement segmentation. Table 1 summarizes the number of computed movements *M* for each subject. In the analysis of activities of daily living, we obtained a varying amounts of movements *M* for each participant as a result of path-based movement segmentation. Table 1 summarizes the total number of computed movements *M* for each individual subject.

**Figure 10:**
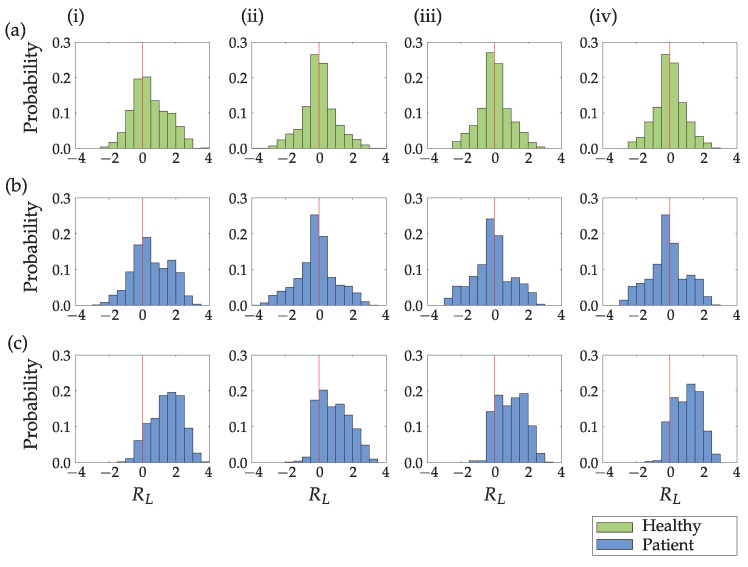
The natural logarithm of the ratio of the hand path lengths of the unaffected (dominant) upper limb and the affected (non-dominant) upper limb for different segmentation methods: path-based segmentation (**i**), and time-based segmentation into one (**ii**), three (**iii**) and five (**iv**) second movement periods for a healthy person (**a**) and two patients after stroke (**b**,**c**). The red vertical line marks the boundary between the unaffected (dominant) and affected (non-dominant) upper limb.

Figure 10 compares movement lengths obtained from path-based and time-based segmentation. Results are presented in Figure 10 (i) as the natural logarithm of the ratio of the hand path lengths for a completed movement in the case of path-based segmentation. Figure 10 (ii)–(iv) present the natural logarithm of the ratio of the hand path lengths with the movement segmented into one-, three- and five-second movement periods (time-based segmentation). Values close to zero indicate similar hand movement lengths of the unaffected (dominant) and affected (non-dominant) upper limbs. Positive values indicate longer movements of the unaffected (dominant) limb compared to the affected (non-dominant) upper limb, and the opposite applies to the negative values.

While time-based segmentation results in symmetric Gaussian-like histograms for the healthy subject and the patient with better upper limb function, the path-based segmentation indicates a shift toward the unaffected (dominant) limb. There are no significant differences between different periods for time-based movement segmentation. In the case of the patient with the reduced upper limb function (Figure 10c) all methods indicate a shift toward the unaffected limb. However, the probability distribution differs between path-based and time-based segmentation.

Results for all participants are summarized in a numerical form in Table 2. We assumed the Gaussian distribution of data presented in Figure 10 and computed the following three parameters: the mean value μ of data, standard deviation σ, and kurtosis κ as a measure of tailedness.

**Figure 11:**
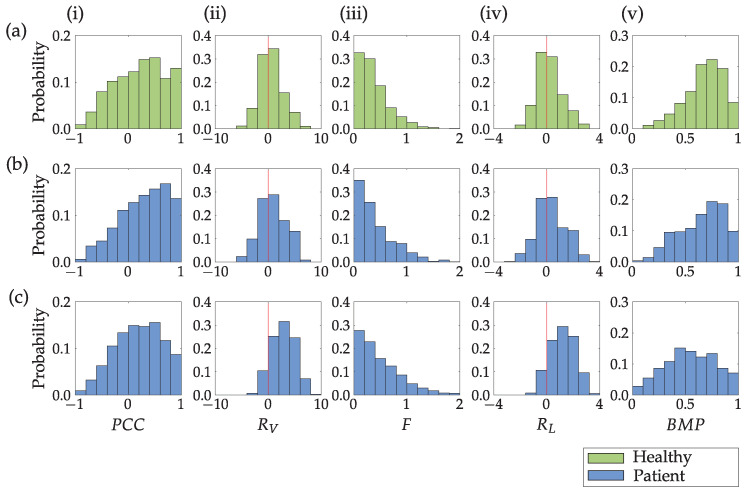
Column (**i**) presents the linear correlation coefficient PCC, (**ii**) the ratio of variances $R_{V}$, (**iii**) Fréchet distance *F*, (iv) the ratio of the hand path lengths $R_{L}$ and (**v**) upper limb bimanual movement parameter BMP, for a healthy person (**a**) and two stroke patients (**b**,**c**). The red vertical line marks the boundary between the unaffected (dominant) and affected (non-dominant) upper limb.

Figure 11 presents the four criteria calculated to assess the bimanual movement coordination of the upper limbs and the bimanual movement parameter BMP that combines those four criteria. The linear correlation coefficient PCC (i), the variance ratio $R_{V}$ (ii), and Fréchet distance *F* (iii) were all computed from the speed profiles of the unaffected (dominant) and affected (non-dominant) upper limbs within the movement *m* resulting from path-based segmentation. The PCC value close to one indicates a significant correlation between the speed profiles. The $R_{V}$ values close to zero indicate similar variances in the speed profiles of the upper limbs. The Fréchet distance *F* was used to evaluate the similarity between the unaffected (dominant) and affected (non-dominant) upper limb speed profiles. A larger Fréchet distance means lower similarity between the curves. The ratio of the hand path lengths $R_{L}$ should be close to zero for similar movement lengths. Higher values of the bimanual movement parameter BMP indicate mostly bimanual upper limbs movement, and the lowest values indicate a unimanual or close to the unimanual movement. All parameters indicate more pronounced bimanual activity for the healthy subject and the patient with better preserved upper limb function compared to the patient with reduced upper limb function.

Table 3 summarizes the distribution of unimanual (BMP<0.4), bimanual BMP>0.7, and unclassified movements (0.4≤BMP≤0.7) for the healthy subject and all patients for the duration of the entire measurement session. The healthy subject and patients with better preserved upper limb functions performed close to 50% of movements bimanually and around 15% unimanually. The ratio is the opposite for the patient with the least preserved upper limb function. The movement length of both upper limbs was approximately equal for bimanual movements and significantly different for unimanual movements. For symmetric movements, shown in Figure 5, 97% of movements were classified as bimanual, 3% were unclassified, and no movements were classified as unimanual.

**Figure 12:**
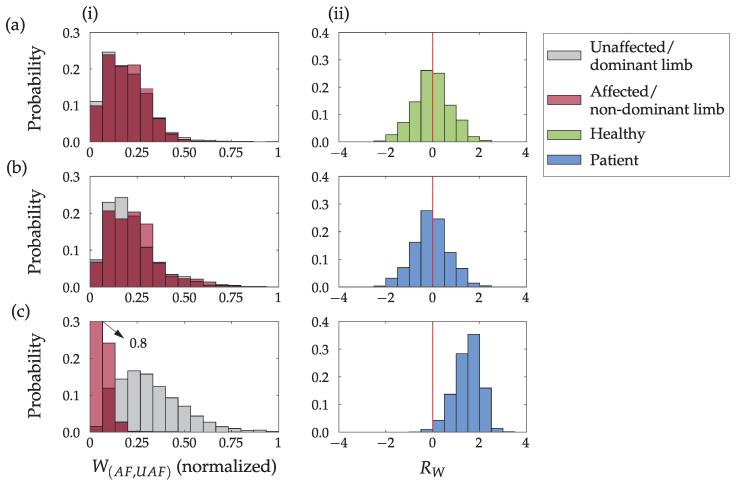
Probability distribution of the normalized activity level for the unaffected (dominant) and affected (non-dominant) upper limbs $W_{\left(AF,UAF\right)}$ (**i**) and the ratio of muscle activities $R_{W}$ (**ii**), for a healthy person (**a**) and two stroke patients (**b**,**c**). The red vertical line marks the boundary between the dominant (unaffected) and non-dominant (affected) upper limb.

Figure 12 presents the muscle activity $W_{\left(AF,UAF\right)}$, computed for each movement *m*. Column 12 (i) presents the normalized muscle potential, and column 12 (ii) presents the ratio of the unaffected (dominant) and the affected (non-dominant) limb muscle potential $R_{W}$. Higher values in Figure 12 (i) indicate a higher level of muscle activity. In Figure 12 (ii), values close to 0 indicate similar muscle activity amplitudes of both upper limbs, higher values imply higher muscular activity of the unaffected (dominant) limb, and negative values imply higher muscular activity of the affected (non-dominant) limb. Highly symmetric activation of forearm muscles was observed for the healthy subject and the patient with better preserved upper limb functions. On the other hand, patient with significant upper limb disability shows highly asymmetric activation. The activation of the affected limb was limited to low EMG amplitudes.

Results for all participants are summarized in a numerical form in Table 4 as they were for segmentation results (Table 2). We also assumed the Gaussian distribution of data presented in Figure 12 (ii) and computed the following three parameters: the mean value μ of data, standard deviation σ, and Kurtosis κ as a measure of tailedness.

## 4. Discussion

Activity counts represent a well-established method for analysis of upper limb activities combined with time-based segmentation and are computed from bandpass-filtered accelerometer data. Comparison of different approaches for the computation of activity counts indicates that when gravitational acceleration was not subtracted from the arm acceleration vector, a greater spread of upper limb activity quants at higher intensities was obtained as shown in Figure 8 (i), compared to the approach with the subtracted gravitational acceleration presented in Figure 8 (ii) and the approach with the subtracted trunk acceleration shown in Figure 8 (iii). Thus, without subtracting the gravitational acceleration, the activity of the upper limbs is incorrectly estimated, as part of the computed activity can be attributed to the gravitational acceleration. The difference between subtracting gravitational acceleration (Figure 8 (ii)) and subtracting trunk acceleration (Figure 8 (iii)) is less evident at first glance, which suggests that trunk activity does not contribute significantly to the assessment of upper limb activity. However, in particular, for the more impaired patient (Figure 8c), the minimal activity of the affected limb can be noticed in column (iii), which cannot be detected without taking into account the movement of the trunk as shown in column (ii). Results in Figure 8a,b do not indicate significant differences between the healthy subject and the less impaired patient. We can observe the smaller intensity of movements for the less impaired patient but no relevant asymmetry. For a more impaired patient, activity quants are shifted to the right (Figure 8c), indicating that the unaffected upper limb is significantly more active, which can be interpreted as predominantly unimanual movement. Most of the unimanual or bimanual activity (red colour in the duration scale) of all three subjects was performed with lower intensity.

The time-based segmentation and the corresponding activity counts enable comparison between the activation of both upper limbs. However, time-based segmentation makes it challenging to analyze functional movements, identify activities, determine movement simultaneity and coordination, observe intentionality, and compute movement lengths. All this is made possible with path-based segmentation into individual completed movements. Additionally, time-based segmentation can, in some cases, produce misleading results. The ratio of simultaneous movement lengths of the dominant to the non-dominant arm shown in Figure 10 confirms this.

The time-based segmentation does not take into account completed movements. Typically, a movement would be split across several consecutive time intervals, introducing randomness in path segmentation. The result of this randomness is a symmetrical probability distribution visible in Figure 10 (ii)–(iv) for the healthy subject and the less impaired patient. This symmetry corresponds to the symmetric distribution of activity counts (Figure 8). The peak of the histogram around value zero indicates that a significant part of the upper limb movements is executed with the same movement length of both upper limbs. Detailed analysis of the time-based segmentation results indicates that the less impaired patient uses his affected arm more than his unaffected arm. The time-based segmentation indicates noticeable asymmetry only for the more impaired patient. However, in this case, the histogram peak is close to value zero (e.g., Figure 10c (ii)). The results of the time-based segmentation indicate that the less impaired patient uses their affected arm more than their unaffected arm. There is also noticeable asymmetry for the more impaired patient, indicating that they primarily use only one hand while the other hand is not active. However, the histogram peak for this patient is close to zero (e.g., Figure 10c (ii)).

On the other hand, the path-based segmentation yields asymmetric results for all subjects (Figure 10 (i)). The probability distributions of arm movement lengths for the healthy subject and the less impaired patient after stroke are similar (Figure 10a,b). The unaffected (dominant) limb typically performs more movements with a longer path than the affected (non-dominant) upper limb. A less impaired patient presented in (b) has no significant asymmetry in path-based segmentation. A more impaired patient (Figure 10c) shows pronounced asymmetry for all four segmentation methods indicating a higher degree of impairment. However, it is important to note that the peak in the histogram for path-based segmentation (Figure 10c (i)) is around value 2. On the other hand, the path-based segmentation shows asymmetry for all subjects (Figure 10 (i)). The probability distributions of arm movement lengths for the healthy subject and the less impaired patient after a stroke are similar (Figure 10a,b). The unaffected (dominant) limb typically performs movements with a longer path than the affected (non-dominant) upper limb. A less impaired patient, presented in (b), has no significant asymmetry in path-based segmentation, but the more impaired patient (Figure 10c) shows pronounced asymmetry in all four segmentation methods, indicating a higher degree of impairment. Importantly, the peak in the histogram for path-based segmentation for the more impaired patient (Figure 10c (i)) is around value 2, indicating that the person only makes short movements with their affected upper limb, performing longer movements primarily with their healthy upper limb. The probability of equal lengths of upper limb movement is only one-third compared with the time-based segmentation. The difference between path-based and time-based segmentation is additionally confirmed by the results for all subjects summarized in Table 2. While there are no significant differences in values for standard deviation σ, the mean value of distribution μ indicates a significant shift toward the unaffected limb for the path-based segmentation compared to the time-based segmentation. The kurtosis κ as a measure of tailedness also confirms differences in probability distribution for the two segmentation methods.

A significant advantage of path-based segmentation, compared to time-based segmentation, is the ability to assess bimanual/unimanual movement and simultaneity. We used four sub-parameters to calculate the bimanual movement parameter BMP and distinguish between three bimanual/unimanual movement levels. For a coordinated bimanual movement of a healthy subject (Figure 5), the average BMP parameter is higher than 0.9, and more than 97% of movements correspond to BMP>0.7 (Figure 6). Considering the representative trajectories shown in Figure 7 for different BMP values, it is possible to confirm strong bimanual activity. With BMP=0.95, the upper limb speed profiles have an almost identical shape. With BMP=0.6, the movements of both limbs are simultaneous but with different intensities, and with BMP=0.25, only one limb is predominantly active.

For a healthy person, we expect the highest proportion of bimanual movements. For bimanual movements (BMP≥0.7), similar movement lengths of both upper limbs are expected. Both hypotheses are confirmed by the results presented in Figure 11 and Table 3 for all subjects. The less impaired patient also performed a significant part of activities bimanually, but the distribution is less shifted to the right compared to the healthy subject. For a more impaired patient, we can observe an almost symmetric distribution between unimanual and bimanual activities (most activities cannot be classified as strictly unimanual or bimanual). A more detailed analysis of movement lengths (Table 3) shows that the average bimanual movement length of the severely affected subject is much shorter than that of the healthy subject or patients with more preserved upper limb functions (0.08 m compared to approximately 0.15 m). For unclassified movements (0.4≤BMP≤0.7), slightly longer movements of the unaffected (dominant) limb were typically observed. However, this cannot be generalized to all subjects. In some cases, movement lengths are equal or even longer movement length of the affected limb can be noted. For predominantly unimanual movements (BMP<0.4), differences in the movement length of both limbs are evident (Table 3).

Often, also the more impaired patients inadvertently or inactively move the upper limb or at least the limb is not at rest. An example of such movement is a passive oscillation of the limb during trunk movement. We did not exclude such movements from the analysis since it is often impossible to distinguish between voluntary and involuntary activity based on trajectory segmentation. However, since these are typically short movements, they do not indicate the functional abilities of the affected upper limb.

We can further reduce the effect of involuntary movements by measuring muscle activity. Muscle activity measured at the forearm is a good indicator of hand activity, such as grasping and manipulating objects. The results in Figure 12 show the differences in muscle activity $W_{\left(AF,UAF\right)}$ of the upper limbs. The differences are negligible for the healthy subject and the less impaired patient, who could efficiently use the affected limb. This is also confirmed by the symmetric distribution of the ratio of the muscle activity parameter $R_{W}$. The symmetric distribution indicates that similar activities were performed with the unaffected (dominant) and the affected (non-dominant) limb. Most often, the healthy subject and the less impaired patients activated left and right forearm muscles at the same time (peak at $R_{W}=0$ in Figure 12, mean value μ≈0 for H, P1 and P2 in Table 4). For a patient with a more impaired upper limb, we measured regular muscle activity in the unaffected upper limb and only low-intensity muscle activity in the affected upper limb. This is also confirmed by the ratio of the muscle activity parameter $R_{W}$ that indicates activation of the forearm muscles on the unaffected limb only (Figure 12). There is almost no simultaneous activation on both arms (with $R_{W}=0$), which is the prevailing pattern for the healthy subject and the less impaired patient. The results of all subjects are summarized in Table 4 with values for standard deviation σ, the mean value of distribution μ and the Kurtosis κ. The mean value of distribution μ indicates a significant shift when comparing the healthy subject (H) and the two patients (P1 and P2) with the less impaired upper limb with the three patients with a more impaired upper limb (P4, P5, and P7). However, there are no relevant differences in the Kurtosis κ or standard deviation σ. These results also support our anticipation of impairment for the patients (P1–P3) as less impaired and patients (P4–P7) as more impaired.

## 5. Conclusions

We implemented a wearable system to monitor and analyze upper limb movement during activities of daily living. This is a crucial step in personalizing the rehabilitation process for patients after a stroke since it enables monitoring of patients outside the clinical environment, thus obtaining objective data on the patient’s condition and progress.

The analysis of upper-limb movement trajectories was performed based on two approaches. In the first approach, we performed time-based segmentation. For each time interval, we computed parameters that determine the activity of the upper limbs: activity counts, movement length, and muscle activity counts. In the second approach, we focused on path-based segmentation for splitting continuous upper limb trajectory into single completed movements, using the methodology validated in [31]. For each completed movement, we computed similar parameters as for time-based segmentation. We showed that path-based segmentation enables a better and more precise insight into the movement capabilities of upper limbs regardless of movement parameters being computed from the movement trajectory or muscle activity. Additionally, path-based segmentation enables functional assessment of upper limb movement. Thus, we proposed the upper limb bimanual movement parameter BMP to estimate the level of bimanual/unimanual movement of upper limbs. The BMP value combines four basic parameters and distinguishes well between unimanual and bimanual movements during the activities of daily living.

When analyzing the results of activities of daily living, some of the ambiguities can be attributed to the fact that we did not distinguish between the time when the subject performed activities sitting at the table or standing. While sitting, the table provides limb weight compensation, thus allowing the patient to use the affected limb more efficiently. A more detailed analysis of the upper limb movement abilities would require data separation into the phases when the limb is supported and when it moves freely in space. Shoulder joint angles could provide valuable information for determining support phases.

Time-based segmentation is a straightforward, real-time approach for identifying trends. It is well-suited for quickly comparing the movements of a unaffected arm to an affected one, and it can provide patients with immediate feedback for using their affected arm more. Additionally, because it does not require detailed kinematic data, it can be implemented using a simpler sensor system, making for more efficient online analysis. Path-based segmentation, on the other hand, is better for more in-depth analysis. It allows for the examination of movement patterns, the range of arm movement, trajectory lengths, and simultaneous movements. However, this method is more complex and would be better suited for offline computation. This type of analysis is important for rehabilitation planning. By using a combination of both methods, it provides a useful tool for both planning and ongoing monitoring of a patient’s activity.

Path-based segmentation opens new possibilities for identifying specific functional uses of upper limbs. Pattern analysis augmented with artificial intelligence approaches could provide an even more in-depth insight into arm performance and enable personalization of upper limb training.

## Figures and Tables

**Table 1:** Number of movements *M* for a healthy participant (H) and stroke patients (P) computed with path-based segmentation method.

	H (a)	P1	P2 (b)	P3	P4 (c)	P5	P6	P7
*M*	1389	1957	1632	1809	2046	2019	1554	2568

**Table 2:** Kurtosis (κ), mean (μ) and standard deviation (σ) for the distribution of ratio $R_{L}$ for a healthy participant (H) and stroke patients (P). Four κ−μ−σ column sets represent different segmentation methods: path-based movement segmentation (column 1), and time-based segmentation into one (column 2), three (column 3) and five (column 4) second movement periods.

			1				2				3				4	
		κ	μ	σ		κ	μ	σ		κ	μ	σ		κ	μ	σ
H (a)		2.6	0.46	1.05		3.7	0.01	0.98		3.3	0.01	0.91		3.3	0.01	0.87
P1		2.8	0.52	1.05		3.5	0.12	1.02		3.3	0.13	0.95		3.2	0.14	0.9
P2 (b)		2.3	0.54	1.13		3.2	−0.14	1.17		2.8	−0.12	1.15		2.7	−0.12	1.12
P3		2.8	0.59	0.94		3.8	0.29	0.84		3.6	0.31	0.76		3.5	0.32	0.72
P4 (c)		2.4	1.45	0.92		2.3	0.92	0.93		2.1	1.00	0.84		2.1	1.03	0.79
P5		2.8	0.96	0.77		2.4	0.63	0.77		2.2	0.68	0.68		2.2	0.70	0.62
P6		2.9	1.00	0.87		3.9	0.62	0.80		3.8	0.65	0.73		3.5	0.67	0.69
P7		3.9	1.78	0.88		2.2	1.01	0.94		2.1	1.11	0.88		2.1	1.18	0.84

**Table 3:** Proportion of bimanual and unimanual movements (%) based on BMP and the median movement lengths of the unaffected (dominant) limb and the affected (non-dominant) limb expressed in meters.

		BMP<0.4 (%)	med($L_{\mathrm{UAF}}$) (m)	med($L_{\mathrm{AF}}$) (m)		0.4≤BMP≤0.7 (%)	med($L_{\mathrm{UAF}}$) (m)	med($L_{\mathrm{AF}}$) (m)		BMP>0.7 (%)	med($L_{\mathrm{UAF}}$) (m)	med($L_{\mathrm{AF}}$) (m)
H (a)		9	0.21	0.06		41	0.16	0.15		50	0.13	0.14
P1		14	0.29	0.09		35	0.20	0.15		51	0.15	0.15
P2 (b)		16	0.33	0.08		36	0.14	0.16		48	0.16	0.17
P3		10	0.24	0.07		34	0.15	0.13		56	0.14	0.13
P4 (c)		28	0.35	0.04		42	0.18	0.09		30	0.11	0.10
P5		14	0.39	0.07		46	0.17	0.11		39	0.12	0.11
P6		19	0.45	0.07		40	0.16	0.10		39	0.13	0.12
P7		43	0.28	0.03		37	0.14	0.07		19	0.09	0.08

**Table 4:** Kurtosis (κ), mean (μ), and standard deviation (σ) for the distribution of ratio of muscle activities $R_{W}$ for a healthy participant (H) and stroke patients (P).

	κ	μ	σ
H (a)	3.02	−0.01	0.77
P1	3.5	0.12	0.73
P2 (b)	3.2	−0.08	0.75
P3	3.2	0.42	0.69
P4 (c)	3.2	1.49	0.55
P5	4.0	1.48	1.0
P6	data were not obtained		
P7	4.4	1.87	0.56
